# Headache Characteristics and Risk Factors Among Healthcare Providers in Al-Ahsa, Saudi Arabia

**DOI:** 10.7759/cureus.45377

**Published:** 2023-09-16

**Authors:** Hassan M Alturaiki, Mohammed A Aldawood, Fatimah Alghirash, Abdullah M Alhajji, Abdullah Almubarak, Shima Al Boesa, Faisal Hakami, Nora AlMuslim

**Affiliations:** 1 College of Medicine, King Faisal University, Al-Ahsa, SAU; 2 Medicine, General Practice, Al-Ahsa, SAU; 3 College of Medicine, Jazan University, Jazan, SAU; 4 College of Medicine, Imam Abdulrahman Bin Faisal University, Dammam, SAU; 5 Neurology, University of Dammam, Dammam, SAU; 6 Neuroimaging, King’s College London, London, GBR

**Keywords:** tension-type headaches, healthcare providers, saudi arabia, alahsa, health care givers, headache

## Abstract

Introduction

Headache is a considerable factor in decreased productivity and work efficiency. This study aims to measure the characteristics of headaches and the risk factors among healthcare providers in Al-Ahsa, Saudi Arabia.

Methods

This cross-sectional study included 353 healthcare providers from Al-Alhsa, Saudi Arabia. An online questionnaire was conducted to assess the demographic and occupational characteristics, headaches, numerous factors such as smoking, caffeine intake, physical activity, and medical conditions to determine their associations with headaches, as well as the impact of headaches on productivity.

Results

Among participants who reported headaches, 15.6% and 4.5% had been previously diagnosed with tension headaches and migraines, respectively. The mean duration of headaches was 7.09±18.16 hours; 38.5% of headaches were throbbing in nature, while 61.5% were pressing. The headache was on one side in 51.6% and accompanied by nausea and vomiting in 41.1% and 24.1%, respectively. Moreover, 53% and 41.9% experienced worsening of headaches by exercise and light, respectively. Tension headaches occurred more in older healthcare providers (P <0.05) and those who reported working night shifts (P=0.002). Healthcare providers with tension headaches reported having an intrusive leader and violence at work (P=0.038 and P=0.013, respectively). Caffeine consumption, emotional stress, and using oral contraceptive pills (OCP) were significantly correlated with migraine (P=0.023, P=0.017, and P=0.004, respectively). The reported headache affected the productivity of 62.59% of individuals.

Conclusions

Headache is common among healthcare providers in Al-Ahsa, Saudi Arabia. The study indicates that tension headache is associated with aging, night shifts, having an intrusive leader, and violence at work. In contrast, migraine is correlated with caffeine consumption, emotional stress, and OCP use. The productivity of healthcare providers is affected negatively by headaches.

## Introduction

Headache is one of the most frequent causes for people to attend neurology clinics in the world. Headache is classified according to the aetiology into primary headache and secondary headache. Primary headache is not a symptom or a consequence of any other disease, the pain itself is the disease, and includes migraine, tension-type headache, and cluster headache. In contrast, secondary headache is not a disease in itself, but a symptom of more serious conditions that occur secondarily to a long list of other conditions. The most common secondary headache is medication-overuse headache. Fortunately, only a small minority of patients whose chief complaint is headache have secondary headaches; most individuals who visit their healthcare practitioner for an examination have a primary headache condition [1]. The World Health Organization (WHO) reports that 1.7-4.0% of individuals experience headaches for 15 or more days every month worldwide [2]. In Saudi Arabia, 65.8% of the population suffers from headaches [1]. The pathophysiology of primary headache syndromes is not fully understood. However, migraine and cluster headaches are thought to be induced by neurological brain dysfunction, with cranial vessel and trigeminal nerve involvement. The parasympathetic nervous system is overactive in the majority of people who suffer from cluster headaches. Overactivity in the cervical or pericranial muscles causes tension-type headaches, which can be caused by neck trauma, poor posture, or anxiety [3-5]. 

Some epidemiological studies have shown that several risk factors, including sex, body mass index (BMI), smoking, family history, climate, excessive caffeine consumption, age, overuse of medication, psychiatric comorbidities (e.g., anxiety, insomnia, and depression), occipital spur, and temporomandibular disorders increased susceptibility to chronic headache [6-9]. Chronic dehydration has been identified as a primary risk factor for headaches in several studies. Sleep deprivation and heavy sleep can cause severe and chronic headaches, which can be avoided by getting seven to nine hours of sleep every night [10]. People who are predisposed to chronic headaches may benefit from reducing their intake of histamine-rich foods (e.g., cheese and other fermented foods) [11]. Tension or migraine headaches can be relieved with the use of sweet pea essential oil and lavender tea [12]. According to several studies, headaches can negatively influence an individual’s life occupationally, financially, sexually, mentally, socially, and emotionally [13-15]. Furthermore, headache has a noticeable association with a decrease in the attendance, ability, and productivity of the employees at work [15-18]. Therefore, headache is considered a cause of disability according to WHO [15].

The purpose of this study is to investigate the prevalence of primary headaches and factors associated with headaches among healthcare providers in Al-Ahsa, Saudi Arabia. Headache has a major negative impact on healthcare workers' productivity and work efficiency. Healthcare workers confront heavy workloads and experience states of high tension and high intensity for long periods. Several studies have shown that primary headache disorders in both nurses and doctors are higher than those in the general population [9]. Therefore, more attention should be paid to the health status of medical workers. In this present study, we aim to assess the characteristics and risk factors of headaches among healthcare providers in Al-Ahsa, Saudi Arabia.

## Materials and methods

This was a descriptive cross-sectional study conducted in Al-Ahsa, Saudi Arabia, from November 2022 to June 2023. The targeted population was primary healthcare providers including physicians and nurses, aged between 20-60 years, and currently living in Al-Ahsa, Saudi Arabia. Any participant not fulfilling the inclusion criteria was excluded. The study was approved by the Research Ethics Committee at King Faisal University (approval number: KFU-REC-2022-NOV-ETHICS318). All the information in this study was used for scientific purposes only, and the participants' confidentiality was a priority.

The sample size was 365 and was calculated using the Richard Geiger equation. We used a self-administrated validated questionnaire that was generated by the study researchers. The questionnaire had two sections: the first section included demographic questions (city, age, gender, marital status, and qualification), and the second section included questions on headaches and associated risk factors.

There were 14 questions related to headache diagnosis and 22 questions related to associated possible risk factors and triggers of headache by its different types.

The electronic questionnaire was distributed to healthcare providers and they were encouraged to share the questionnaire with their colleagues using a snowball sampling technique. The data was entered into Microsoft Excel 2016 (Microsoft Corporation, Redmond, Washington, United States). The statistical analysis was conducted using IBM SPSS Statistics for Windows, Version 25.0 (Released 2017; IBM Corp., Armonk, New York, United States). Chi-square test was used to analyze the data. Statistical significance was defined as p-value of less than 0.05, with a confidence interval of 95%.

## Results

Sociodemographic characteristics

The survey was completed by a total of 353 participants who were included in the analysis. All the participants experienced headaches. The majority of these individuals were females (65.2%) and were in their 20s and 30s (65.7%). Less than half of the participants were married (43.6%), and two-thirds held bachelor's degrees (66.6%). Moreover, physicians had the highest response rate (38%) while nurses had the lowest response rate (22.9%) as given in Table 1. Smoking and caffeine consumption were reported by 12.5% and 67.1% of participants, respectively. A smaller percentage of participants reported experiencing anxiety (11.3%), depression (8.5%), and obesity (10.5%). Over half (55.5%) engaged in regular physical activity, and the majority (70.8%) did not use nonsteroidal anti-inflammatory drugs (NSAIDs), oral contraceptives (OCPs), or other medications. Emotional stress affected 40.5% of the study participants (Table 2).

**Table 1:** Associations of headache types with socio-demographic characteristics AHP: allied health professionals

Socio-demographic characteristics	Total, n = 353 (100%)	Diagnosed with migraine, n = 16 (4.5%)	Diagnosed with tension headache, n = 55 (15.6%)	Diagnosed with another type, n = 5 (1.4%)	Not diagnosed, n = 277 (78.5%)	P-value
Gender						
Male	123 (34.8%)	5 (4.1%)	12 (9.8%)	1 (0.8%)	105 (85.4%)	0.109
Female	230 (65.2%)	11 (4.8%)	43 (18.7%)	4 (1.7%)	172 (74.8%)	
Age groups						
20-30 year old	232 (65.7%)	6 (2.6%)	33 (14.2%)	4 (1.7%)	189 (81.5%)	
31-45 year old	87 (24.6%)	9 (10.3%)	12 (13.8%)	1 (1.1%)	65 (74.7%)	0.033*
>45 year old	34 (9.6%)	1 (2.9%)	10 (29.4%)	0 (0%)	23 (67.6%)	
Marital Status						
Married	154 (43.6%)	11 (7.1%)	19 (12.3%)	1 (0.6%)	123 (79.9%)	0.069
Non-Married	199 (56.4%)	5 (2.5%)	36 (18.1%)	4 (2%)	154 (77.4%)	
Educational level						
Diploma	64 (18.1%)	4 (6.3%)	12 (18.8%)	1 (1.6%)	47 (73.4%)	
Bachelor	235 (66.6%)	9 (3.8%)	32 (13.6%)	3 (1.3%)	191 (81.3%)	0.143
Master	35 (9.9%)	0 (0%)	6 (17.1%)	1 (2.9%)	28 (80%)	
PhD	19 (5.4%)	3 (15.8%)	5 (26.3%)	0 (0%)	11 (57.9%)	
Occupation						
AHP & others	84 (23.8%)	1 (1.2%)	11 (13.1%)	2 (2.4%)	70 (83.3%)	
Nurse	81 (22.9%)	4 (4.9%)	10 (12.3%)	1 (1.2%)	66 (81.5%)	0.371
Physician	134 (38%)	10 (7.5%)	22 (16.4%)	2 (1.5%)	100 (74.6%)	
Pharmacist	54 (15.3%)	1 (1.9%)	12 (22.2%)	0 (0%)	41 (75.9%)	

**Table 2:** Association of headache types with personal risk factors NSAIDs: non-steroidal anti-inflammatory drugs; OCP: oral contraceptive pills

Risk factors	Total, n = 353 (100%)	Diagnosed with migraine, n = 16 (4.5%)	Diagnosed with tension headache, n = 55 (15.6%)	Diagnosed with another type, n = 5 (1.4%)	Not diagnosed, n = 277 (78.5%)	P-value
Smoking						
No	309 (87.5%)	13 (4.2%)	44 (14.2%)	5 (1.6%)	247 (79.9%)	0.185
Yes	44 (12.5%)	3 (6.8%)	11 (25%)	0 (0%)	30 (68.2%)	
Caffeine Consumption						
No	116 (32.9%)	1 (0.9%)	25 (21.6%)	1 (0.9%)	89 (76.7%)	0.023*
Yes	237 (67.1%)	15 (6.3%)	30 (12.7%)	4 (1.7%)	188 (79.3%)	
Physically Active						
No	157 (44.5%)	6 (3.8%)	21 (13.4%)	2 (1.3%)	128 (81.5%)	0.661
Yes	196 (55.5%)	10 (5.1%)	34 (17.3%)	3 (1.5%)	149 (76%)	
Depression						
No	323 (91.5%)	16 (5%)	48 (14.9%)	4 (1.2%)	255 (78.9%)	0.227
Yes	30 (8.5%)	0 (0%)	7 (23.4%)	1 (3.3%)	22 (73.3%)	
Anxiety						
No	313 (88.7%)	12 (3.8%)	47 (15%)	5 (1.6%)	249 (79.6%)	0.204
Yes	40 (11.3%)	4 (10%)	8 (20%)	0 (0%)	28 (70%)	
Obesity						
No	316 (89.5%)	14 (4.4%)	46 (14.6%)	4 (1.3%)	252 (79.7%)	0.217
Yes	37 (10.5%)	2 (5.4%)	9 (24.3%)	1 (2.7%)	25 (67.6%)	
Drug-Related						
NSAIDs	63 (17.8%)	8 (12.7%)	14 (22.2%)	2 (3.2%)	39 (61.9%)	
OCP	31 (8.8%)	0 (0%)	8 (25.8%)	0 (0%)	28 (74.2%)	0.004*
Taking both NSAID and OCP	9 (2.5%)	0 (0%)	3 (33.3%)	0 (0%)	6 (66.7%)	
Others or not using medications	250 (70.8%)	8 (3.2%)	30 (12%)	3 (1.2%)	209 (83.6%)	
Emotional Stress						
No	210 (59.5%)	5 (2.4%)	37 (17.6%)	5 (2.4%)	163 (77.6%)	0.017*
Yes	143 (40.5%)	11 (7.7%)	18 (12.6%)	0 (0%)	114 (79.7%)	

Table 3 presents various work-related factors. Only 21.8% of participants had intrusive leaders in their work setting, while 26.6% and 24.1% frequently worked overtime and received low work rewards, respectively. High work effort was reported by 34.6% of participants, and 33.4% had worked for seven years or more. Most participants (83%) worked in quiet environments, and 40.4% did not work night shifts.

**Table 3:** Association of headache types with work-related factors

Work-related factors	Total, n = 353 (100%)	Diagnosed with migraine, n = 16 (4.5%)	Diagnosed with tension headache, n = 55 (15.6%)	Diagnosed with another type, n = 5 (1.4%)	Not diagnosed, n = 277 (78.5%)	P-value
Intrusive leader/boss						
No	276 (78.2%)	11 (4%)	36 (13%)	5 (1.8%)	224 (81.2%)	0.038*
Yes	77 (21.8%)	5 (6.5%)	19 (24.7%)	0 (0%)	53 (68.8%)	
Requirement of working overtime						
No	259 (73.4%)	13 (5%)	42 (16.2%)	4 (1.5%)	200 (77.2%)	0.865
Yes	94 (26.6%)	3 (3.2%)	13 (13.8%)	1 (1.3%)	77 (81.9%)	
Exerted high effort						
No	231 (65.4%)	10 (4.3%)	30 (13%)	3 (1.3%)	188 (81.4%)	0.264
Yes	122 (34.6%)	6 (4.9%)	25 (20.5%)	2 (1.6%)	89 (73%)	
Low work-related rewards						
No	268 (75.9%)	12 (4.5%)	40 (14.9%)	5 (1.9%)	211 (78.7%)	0.711
Yes	85 (24.1%)	4 (4.7%)	15 (17.6%)	0 (0%)	66 (77.6%)	
Work duration of 7 years and more						
No	235 (66.6%)	6 (2.6%)	35 (14.9%)	4 (1.7%)	190 (80.9%)	0.068
Yes	118 (33.4%)	10 (8.5%)	20 (16.9%)	1 (0.8%)	87 (73.7%)	
Violence at work						
No	293 (83%)	14 (4.8%)	37 (12.6%)	5 (1.7%)	237 (80.9%)	0.013*
Yes	60 (17%)	2 (3.3%)	18 (30%)	0 (0%)	40 (66.7%)	
Night shifts (per month)						
Less than 6	105 (29.7%)	4 (3.8%)	27 (25.7%)	2 (1.9%)	72 (68.6%)	
More than 6	102 (28.7%)	18 (17.6%)	4 (3.9%)	1 (1%)	79 (77.5%)	0.002*
Not applicable	146 (41.4%)	10 (6.8%)	8 (5.5%)	2 (1.4%)	126 (86.3%)	

Types of headaches and their associations with participants' characteristics, work-related factors, and personal risk factors

In the 353 participants, headaches were categorized into physician-diagnosed migraines (4.5%), tension headaches (15.6%), and other types (1.4%), with a large portion remaining undiagnosed (78.5%). In our analysis, we examined the relationship between different types of headaches and diagnostic status. Using statistical tests like Person Chi-Square and Fisher's Exact tests, we did not find any significant associations between headache types and demographic characteristics. However, we did observe that 81.5% of participants who had headaches but were not diagnosed by a physician were between the ages of 20-30. Additionally, participants above the age of 45 were more likely to be diagnosed with tension headaches compared to others. Therefore, age was the only demographic variable that showed significance. The association of headache types with socio-demographic characteristics and personal risk factors is shown in Tables 1-2.

Participants consuming caffeine, using OCPs, and experiencing emotional stress were significantly more likely to be diagnosed with migraines (P = 0.02, P = 0.004, and P = 0.01, respectively). Those having intrusive leaders and working in violent environments had a higher likelihood of being diagnosed with tension-type headaches (P = 0.03, P = 0.01). Night shifts were significantly associated with tension headaches (P = 0.002). The association of headaches with work-related factors is given in Table 3.

Headache characteristics among the subjects

**Figure 1:**
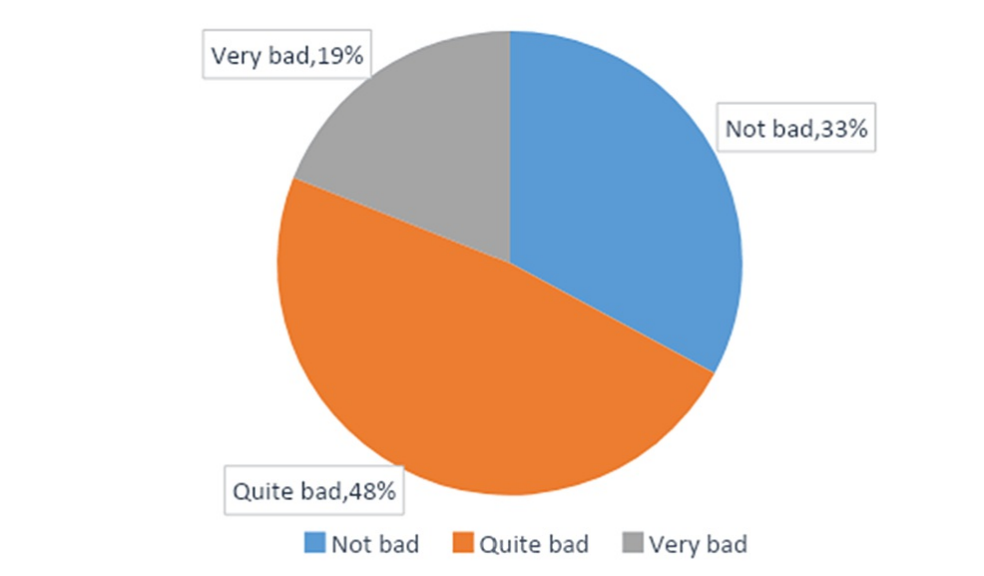
How bad the pain is?

**Figure 2:**
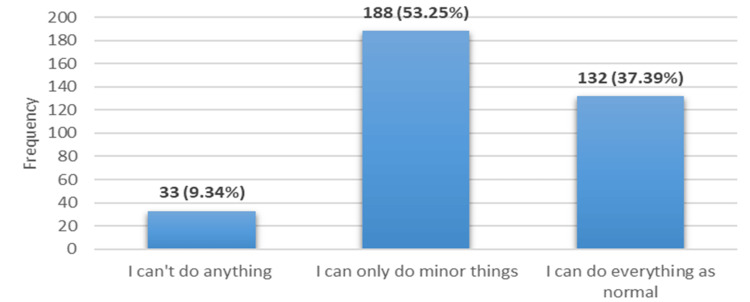
How does the headache affect your ability to do day-to-day activity?

Table 4 shows that the average headache duration was 7.09 hours, with a standard deviation of 18.16 hours. Approximately one-fifth of participants experienced headaches more than 15 times per month. Participants described their headache pain as pulsating or throbbing (38.5%) or tightening or pressing (61.5%). About half (51.6%) reported one-sided pain of the head and worsening with exercise (51.6% and 53%, respectively). Nausea and vomiting were reported by 41.1% and 24.1% of participants, respectively, and 41.9% said their headaches worsened with exposure to light. Among female participants, 62.17% reported headaches coinciding with their menstrual cycles. One-third of participants described their headaches as not very severe (Figure 1), while a small percentage (9.34%) reported that headaches significantly impacted their lives, rendering them unable to function normally (Figure 2).

**Table 4:** Headache characteristics Data given as n (%) unless otherwise mentioned

Headache Characteristics	n, (%)
Headache Duration (per hour), mean±SD (minimum-maximum)	7.09±18.16 [0.10-168]
Headache Frequency (per month)	
Less than 2 times	93 (26.3%)
More than 2 times, but less than 8 times	123 (34.8%)
More than 8 times, but less than 15 times	66 (18.7%)
More than 15 times	71 (20.1%)
Throbbing or Pulsating Headache	136 (38.5%)
Tightening or Pressing Headache	217 (61.5%)
One Sided Pain	182 (51.6%)
Worsen by Exercise	187 (53%)
Associated with the Menstrual Cycle	143 out of 230 (62.17%)
Associated with Nausea	145 (41.1%)
Associated with Vomiting	85 (24.1%)
Worsened by Light	148 (41.9%)

## Discussion

This study investigated the associations between headache types and socio-demographic characteristics. Regarding gender, our results showed that there was no statistically significant association between gender and headache types. This finding is consistent with previous studies, which also reported no significant gender differences in the prevalence of headache types [19]. However, it is worth noting that a higher proportion of females were diagnosed with tension headaches compared to males, although this association did not reach statistical significance. This trend aligns with the findings of previous research, that observed a higher prevalence of tension headaches among females [19].

In terms of age groups, we found a statistically significant association between age and headache types. Specifically, a higher proportion of individuals diagnosed with tension headaches were in the older age group (above 45 years old). This result is consistent with a study conducted in 2014, which identified a higher prevalence of tension headaches in older age groups [20]. However, for other headache types such as migraine and another type of headache, no significant associations were observed across age groups. These findings contrast with the study by Kelman et al., which reported significant age-related differences in the prevalence of migraine and other headache types [21].

Regarding marital status, our study did not find a statistically significant association between marital status and headache types. This finding is in line with previous research conducted by Winter et al., which similarly reported no significant associations between marital status and headache types [22]. However, it is interesting to note that a higher proportion of individuals diagnosed with tension headaches were unmarried, although this association did not reach statistical significance.

In terms of educational level, our results did not reveal a statistically significant association between educational level and headache types. This finding is consistent with the study by Almalki et al., which also reported no significant associations between educational level and the prevalence of different headache types [3].

Regarding headache duration, the mean duration reported by participants was 7.09 hours, with a notable standard deviation of 18.16 hours. This suggests a significant variability in the duration of headaches within the studied population. The range of headache duration was found to vary widely, ranging from 0.10 to 168 hours. These findings align with previous studies that have highlighted the heterogeneity of headache duration [23]. It is important to note that the reported mean duration might be influenced by individual variations, headache types, and potential comorbidities.

Headache frequency analysis revealed that a substantial proportion of participants experienced headaches more than twice a month but less than eight times a month (34.8%). Moreover, 26.3% reported experiencing headaches less than two times a month, while 18.7% experienced headaches more than eight times but less than 15 times a month. The remaining 20.1% of participants reported experiencing headaches more than 15 times a month. These findings are consistent with previous studies that have reported varying headache frequencies in different populations [24].

In terms of headache characteristics, the study found that the majority of participants experienced tightening or pressing headaches (61.5%), while a considerable proportion reported throbbing or pulsating headaches (38.5%). These results are in line with previous studies that have identified tension-type headaches as the most prevalent type [25]. Additionally, a significant number of participants reported one-sided pain (51.6%), headaches worsened by exercise (53%), and an association between headaches and the menstrual cycle (62.17%). These findings align with existing literature that has established the association between these characteristics and certain headache types, such as migraine [26].

The study also explored symptoms associated with headaches. A notable proportion of participants reported experiencing nausea (41.1%) and sensitivity to light (41.9%), while fewer participants reported vomiting (24.1%). These findings are consistent with previous studies that have identified these symptoms as common accompanying features of various headache types [27].

Furthermore, the study assessed the impact of headaches on participants’ daily activities. The results indicated that a small portion of participants reported being unable to perform any activities during headache episodes (9.34%). However, a majority of participants reported being able to carry out minor tasks (53.25%), while a smaller proportion reported no interference with their daily activities (37.39%). These findings are consistent with previous research highlighting the substantial impact of headaches on individuals’ ability to perform daily tasks [27].

The study examined the severity of pain experienced during headaches. The majority of participants reported the pain as quite bad (48%), followed by those who considered it not bad (33%) and very bad (19%). These findings align with previous studies that have reported varying degrees of pain severity in headache populations [28].

Our study did not find a statistically significant association between occupation and headache types. This result aligns with previous studies conducted by Di Prinzio et al., which similarly reported no significant associations between occupation and the prevalence of headache types [29]. However, it is noteworthy that a higher proportion of individuals diagnosed with tension headaches were pharmacists, although this association did not reach statistical significance.

The association between smoking and headache types was explored, and the results indicated that individuals who reported smoking had a slightly higher prevalence of migraine and tension headaches compared to non-smokers. However, the association was not statistically significant (P = 0.185). However, Waldie et al. found a significant association between smoking and tension headache [30], which contrasts with our results. The lack of statistically significant results in our study could be due to the small sample size or other unmeasured factors.

Our findings revealed a significant association between caffeine consumption and headache types (P = 0.023). Specifically, individuals who reported caffeine consumption had a higher prevalence of migraine and tension headaches compared to those who reported no caffeine consumption. These results align with previous studies, which found a positive association between caffeine intake and migraine [31]. The stimulant properties of caffeine and its effects on blood vessels and neurotransmitters may contribute to the development of headaches. However, it should be noted that the amount of daily caffeine consumption wasn't investigated in our current study.

The association between physical activity and headache types did not reach statistical significance in our study (P = 0.661). Both physically active and inactive individuals exhibited similar prevalence rates of migraine, tension headache, and other types. These results are consistent with previous research by Lippi et al., which did not find a significant association between physical activity and migraine or tension headache [32]. However, it is important to note that the definition and measurement of physical activity may vary across studies, and other factors such as exercise intensity and frequency might influence the association.

Our study did not find a significant association between depression and headache types (P = 0.227). Individuals with and without depression showed comparable prevalence rates of migraine, tension headache, and other types. These results differ from a study conducted by Janke et al., which reported a significant association between depression and both migraine and tension headaches [33]. This discrepancy could be due to differences in the sample characteristics, such as the age, gender, or occupation of the participants. It could also be due to variations in the way that overtime was assessed.

Similar to depression, anxiety did not exhibit a statistically significant association with headache types (P = 0.204). Individuals with and without anxiety had comparable prevalence rates of migraine, tension headache, and other types. These results contrast with a previous study by Kumar et al., which identified a positive association between anxiety and migraine [34]. This might be due to differences in the assessment of anxiety or sample characteristics.

The association between obesity and headache types was not statistically significant in our study (P = 0.217). Both non-obese and obese individuals showed similar prevalence rates of migraines, tension headaches, and other types of headaches. However, Verrotti et al. reported a positive association between obesity and tension headache [35], contradicting our results. Discrepancies among studies might arise from differences in sample characteristics, the definition of obesity, or unaccounted confounding factors.

We examined the associations between specific drug-related factors and headache types, including nonsteroidal anti-inflammatory drugs (NSAIDs) use, oral contraceptive pill (OCP) use, and a combination of both. Among the drug-related factors, only OCP use showed a significant association with tension headache (P = 0.004). Individuals using OCP had a higher prevalence of tension headaches compared to non-users. Our findings align with a study by Allais et al., which reported a positive association between OCP use and headache [36].

We explored the association between emotional stress and headache types, and the results indicated a significant association (P = 0.017) between emotional stress and migraines. Individuals reporting emotional stress had a higher prevalence of migraines compared to those without stress. These findings are consistent with previous studies [37]. However, no significant association was found between stress and tension headaches or other types in our study. It is important to note that the experience and perception of stress can vary among individuals, and additional factors may contribute to headache development.

The results indicate that individuals with no headache diagnosis reported the highest percentage (78.2%), followed by those diagnosed with tension headaches (13%) and other types of headaches (1.8%). Interestingly, individuals diagnosed with migraines had a significantly lower percentage (4%) compared to the other groups. This finding suggests a potential association between an intrusive leader or boss and tension headaches. However, it is important to note that the association between an intrusive leader/boss and migraines was not statistically significant (P = 0.038). For instance, Lin et al. conducted a similar study and reported a significant association between work-related stress and tension-type headaches [38]. Their findings support the current study’s results, indicating that an intrusive leader or boss may contribute to the development of tension headaches.

In this study, the requirement of working overtime did not have a significant association with the occurrence of headaches (P = 0.865). The percentage of individuals diagnosed with migraines and tension headaches remained relatively stable across different overtime requirements. A study by Leso et al. examined the relationship between long working hours and headache occurrence [39]. Their findings suggested that long working hours were associated with an increased risk of headaches, including migraines. However, the current study did not find a significant association between overtime requirements and headaches. This discrepancy might be due to differences in sample characteristics or variations in the assessment of overtime.

The analysis revealed that there was no statistically significant association between exerting high effort at work and the occurrence of headaches (P = 0.264). The percentage of individuals diagnosed with tension headaches was slightly higher in the “diagnosed with headache” group compared to the “no diagnosis” group. However, the difference was not significant enough to establish a conclusive relationship. It is in contrast to prior studies, indicating that high effort at work was significantly associated with headaches.

The results demonstrate that there was no significant association between low work-related rewards and the occurrence of headaches (P = 0.711). The percentages of individuals diagnosed with migraines and tension headaches in both the “no diagnosis” and “diagnosed with headache” groups remained relatively consistent across different levels of work-related rewards. In contrast to these findings, a study by Aazami et al. found that low job satisfaction and inadequate rewards were significantly associated with an increased risk of headaches [40].

Regarding the work duration, there was no statistically significant association between a work duration of seven years or more and the occurrence of headaches (P = 0.068). The percentage of individuals diagnosed with migraines and tension headaches in both the “no diagnosis” and “diagnosed with headache” groups did not differ significantly across different work durations. In a study conducted by Sato et al., they investigated the relationship between work duration and headache prevalence. The study found that people who worked longer hours were more likely to develop headaches, including migraines [41]. However, the current study did not find a significant association between a work duration of seven years and more and headaches. This discrepancy could be due to variations in the definition of work duration or differences in the study populations.

Interestingly, our finding revealed a significant association between violence at work and the occurrence of tension headaches (P = 0.013). The percentage of individuals diagnosed with tension headaches was significantly higher. However, it is important to note that the association between violence at work and migraines was not statistically significant. Previous studies have explored the impact of workplace violence on headaches and related symptoms. For instance, a study by Magnavita et al. (2022) found a significant association between workplace violence and both migraines and tension headaches [42]. Their findings align with the current study’s results, suggesting that experiencing violence at work may contribute to the development of tension headaches.

Finally, the results indicate a significant association between the number of night shifts per month and the occurrence of tension headaches (P = 0.002). The percentage of individuals diagnosed with migraines was significantly higher for both the “less than six” and “more than six” night shifts categories. These results are in line with an earlier study by Sandoe et al., who found a significant link between working night shifts and an elevated risk of migraines [43]. Migraines may occur as a result of night shift work's disturbance of the circadian rhythm and sleep cycles.

The limitations of this study are the small sample size, the possibility of recall bias arising from patients’ answers, and the coverage of a single city. Despite investigating the lifestyle factors, such as caffeine consumption, smoking, and lack of exercise that could affect the headache characteristics, we did not investigate them in detail. Further research will be appreciated.

## Conclusions

Our study shows the prevalence of primary headache disorders (including migraine and tension headaches) in healthcare providers in Al-Ahsa, Saudi Arabia. The study showed no significant between headache types and socio-demographic characteristics or gender. Multiple factors significantly associated with migraine and tension headaches in healthcare providers were verified, including ageing, night shifts, having an intrusive leader, and violence at work. Multiple factors were insignificant, including caffeine consumption, emotional stress, marital status, educational level, and physical activity. To improve the health of healthcare providers, headache education and strategies for managing these factors should be effectively implemented.
